# Clinical Implications of Immune Dysfunction in Chronic Lymphocytic Leukemia

**DOI:** 10.3390/cancers18091323

**Published:** 2026-04-22

**Authors:** Luis Miguel Juárez-Salcedo, Javier Loscertales

**Affiliations:** 1 Hematology Department, La Princesa University Hospital, 28006 Madrid, Spain; 2 Human Medicine Department, Autonoma University of Madrid, 28049 Madrid, Spain

**Keywords:** immunosuppression, infections, immune cytopenias, immune checkpoints, second malignancies

## Abstract

The immunosuppression inherent to chronic lymphocytic leukemia (CLL) is the most frequent cause of morbidity and mortality associated with this pathology. Both the innate and adaptive immune responses exhibit marked functional alterations, with a bias towards a tolerant environment that favors the spread of the disease. This condition is reflected in increased risk of infections, immune-mediated cytopenias, and associated second malignancies. Knowledge of these alterations, both in the molecular pathways that modulate T cell activity in CLL (the T lymphocyte cytotoxic antigen-4 (CTLA-4) axis and programmed cell death 1 (PD-1)) and at the T cell immunoreceptor level, could be of interest as therapeutic targets in CLL. In this review, we will analyze the main consequences of this dysfunction and its management strategies.

## Simple Summary

Chronic lymphocytic leukemia (CLL) is a malignant neoplasm of B lymphocytes characterized by profound and persistent immune dysfunction. This review summarizes how defects affecting both innate and adaptive immunity contribute to the major clinical complications of CLL, including infections, autoimmune cytopenias, and secondary neoplasms. These patients exhibit impaired antibody production, dysfunctional T-cell responses, and impaired innate immune cell function, leading to increased susceptibility to infections. Furthermore, immune dysregulation promotes the development of autoimmune cytopenias and reduces tumor immune surveillance, thereby facilitating the emergence of secondary neoplasms. Although targeted therapies have improved disease control and can partially restore immune balance, immune dysfunction often persists and continues to affect outcomes. Understanding these mechanisms is essential for optimizing prevention strategies, guiding clinical management, and developing therapies that not only control the disease but also restore immune competence.

## 1. Introduction/Pathophysiology

Chronic lymphocytic leukemia (CLL) is a hematologic neoplasm of mature B-lymphocytes characterized by clonal expansion and accumulation of leukemic cells in the peripheral blood, bone marrow, lymph nodes, and spleen [1]. Disease progression and prognosis are determined by the interplay between intrinsic genetic alterations relevant for risk stratification and signals derived from the tumor microenvironment that shape leukemic cell survival and behavior [2].

In addition to these oncogenic features, this disease is characterized by a profound immune dysfunction affecting both innate and adaptive immune responses [3]. This immunodeficiency is an intrinsic and early feature of the disease, progressively worsening over time and largely independent of treatment exposure, and it underlies the increased susceptibility to infections, impaired vaccine responses, autoimmune complications, and secondary malignancies [4].

Adaptive immune dysfunction in CLL is driven by both quantitative and qualitative defects in humoral and cellular immunity. Malignant B cells are functionally incompetent and interfere with normal B-cell differentiation, leading to hypogammaglobulinemia and defective antibody production [5]. In parallel, T cells display significant functional abnormalities despite preserved or increased absolute numbers. These include impaired immune synapse formation, reduced cytotoxic capacity, altered cytokine production, and features of chronic activation and exhaustion. At the molecular level, these defects are associated with dysregulated T-cell receptor signaling, metabolic reprogramming, and overexpression of inhibitory immune checkpoints such as PD-1 and CTLA-4, as well as expansion of regulatory T-cell populations, collectively contributing to impaired antiviral and antitumor immunity and loss of peripheral tolerance [6].

Innate immune responses are similarly compromised. Natural killer cells exhibit reduced cytotoxicity and defective activation, while neutrophils show impaired chemotaxis, phagocytosis, and oxidative burst, resulting in inefficient bacterial and fungal clearance [7]. Monocytes and dendritic cells also display functional defects, including damage antigen presentation and altered cytokine secretion. In addition, deficiencies in the classical complement pathway further weaken opsonization and antibody-mediated immune mechanisms [8].

These immune abnormalities are sustained and amplified by continuous interactions between CLL cells and their microenvironment. The tumor microenvironment is typically hypoxic, as a result of the uncontrolled proliferation of cancer cells and abnormal vascularization [9,10,11]. Hypoxia also enhances glycolytic energy supply (necessary for proliferation) by activating HIF1α-driven transcriptional reprogramming. This positive feedback leads to acidification of the extracellular space due to extracellular lactate accumulation, which affects tumor-associated immune cells and stromal elements, creating a more permissive environment. Increased lactate attenuates the activation of dendritic cells and T cells, inhibits monocyte migration, and promotes the polarization of macrophages toward an M2 phenotype [12,13].

**Figure 1:**
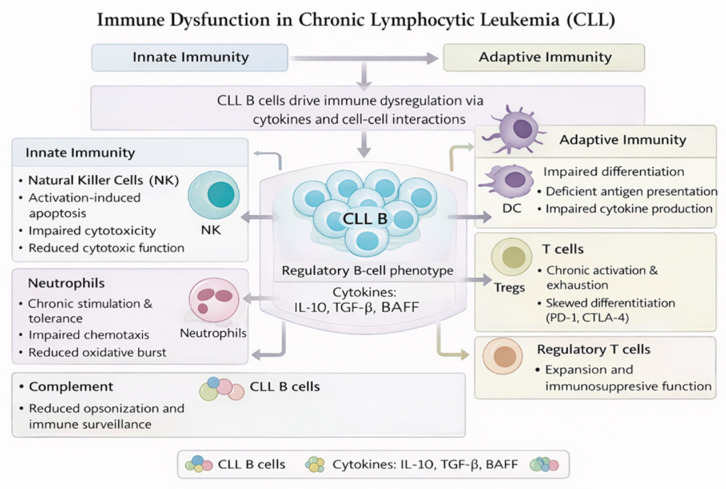
Innate and humoral immune dysfunction in chronic lymphocytic leukemia. Chronic lymphocytic leukemia is characterized by profound alterations of innate and humoral immunity driven by leukemic B cells. Dysfunctional interactions with neutrophils, natural killer cells, dendritic cells, monocytes/macrophages, and the complement system impair immune surveillance and pathogen clearance, promoting immune tolerance and supporting leukemic cell survival.

Leukemic cells secrete immunomodulatory cytokines such as IL-10, TGF-β, and BAFF, which suppress effective immune responses while promoting immune tolerance [14]. Chronic antigenic stimulation and persistent activation of signaling pathways, including B-cell receptor, NF-κB, and PI3K/AKT/mTOR pathways, contribute to immune exhaustion and systemic immune dysregulation [15]. Together, these interconnected defects create a permissive environment that underlies the broad spectrum of infectious, autoimmune, and neoplastic complications observed in patients with CLL (Figure 1).

Rather than representing isolated abnormalities, defects in innate and adaptive immunity in CLL are deeply interconnected and mutually reinforcing. This global immune dysfunction explains the coexistence of infectious susceptibility, autoimmunity, impaired vaccine responses, and increased cancer risk in CLL patients. Importantly, many of these immune defects persist despite effective control of leukemic burden, underscoring the need for therapeutic strategies that address not only tumor eradication but also immune restoration.

In this review, we examine the mechanisms and clinical impact of immune dysfunction in CLL, focusing on infectious susceptibility, autoimmune cytopenias, and secondary neoplasms, and discuss how advances in therapy have reshaped their management while highlighting ongoing unmet clinical needs.

## 2. Infections

Infections represent one of the leading causes of morbidity and mortality in patients with CLL. This elevated susceptibility is not only a consequence of treatment-related immunosuppression but also a hallmark of the disease itself. CLL is characterized by profound, multifactorial immune dysfunction that affects humoral, cellular, and innate immunity, and this impairment evolves dynamically throughout the disease course and in response to therapy [8,16]. As a result, infections may occur at any disease stage, including in previously untreated patients, and remain a major clinical challenge even in the era of targeted therapies [17,18]. Table 1 summarizes the different immune defects and their clinical consequences.

### 2.1. Defects in Humoral Immunity

Humoral immunodeficiency is a central feature of CLL and is among the strongest predictors of infection. The progressive expansion of monoclonal, dysfunctional B cells interferes with normal B-cell development and antibody production, leading to hypogammaglobulinemia in up to 85% of patients over the course of the disease [19,20]. Beyond quantitative immunoglobulin deficiency, qualitative defects in antibody production limit the ability to mount effective responses against encapsulated bacteria such as *Streptococcus pneumoniae* and *Haemophilus influenzae* [21]. These abnormalities contribute to recurrent sinopulmonary infections, sepsis, and ineffective vaccine responsiveness. The diminished immunogenicity of pneumococcal, influenza, and viral vaccines in CLL further increases infection risk, underscoring the importance of vaccination during the early or untreated phases of the disease, when feasible.

Another clear example relates to vaccination against SARS-CoV-2. It has been demonstrated that patients with CLL receiving BTK inhibitors (BTKis), regardless of generation, exhibit a markedly reduced humoral response to mRNA COVID-19 vaccines, with seroconversion rates ranging from 22% to 53% after primary vaccination, compared with approximately 72% in treatment-naïve patients and over 95% in healthy controls [22,23].

Most studies have evaluated covalent BTKis as a class, without consistent direct comparisons between ibrutinib and second-generation agents such as acalabrutinib or zanubrutinib. Across cohorts, BTKi-treated patients exhibit very low antibody titers (e.g., median anti-S1 IgG ~0 AU/mL vs. >5000 AU/mL in untreated patients) [22,24]. Current evidence does not demonstrate a clinically meaningful difference between ibrutinib and second-generation BTKis in vaccine response, suggesting that this impairment represents a class effect.

### 2.2. Dysregulation of Cellular Immunity

In addition to humoral impairment, CLL induces important alterations in T-cell immunity. T cells exhibit features of functional exhaustion, including overexpression of inhibitory receptors, reduced cytotoxic activity, and impaired cytokine production [25]. These changes affect both CD4^+^ and CD8^+^ compartments and compromise antiviral and antitumoral responses. Furthermore, expansion of regulatory T cells (Tregs) contributes to an immunosuppressive microenvironment that promotes leukemia cell survival and limits effective immune surveillance [26]. These abnormalities explain the increased incidence of viral infections—such as herpesvirus reactivation, respiratory viral infections, and opportunistic pathogens—in patients with CLL, even in the absence of therapy [18].

### 2.3. Defects in Innate Immunity

Innate immunity is profoundly altered in CLL, and these defects contribute meaningfully to infectious vulnerability. Neutrophils, in particular, demonstrate substantial functional impairment, including defective migration, reduced phagocytic capacity, and diminished oxidative burst. Natural killer (NK) cells similarly show compromised cytotoxic activity and impaired formation of immunological synapses, limiting their ability to eliminate infected or malignant cells [15].

A striking paradox emerges in the behavior of neutrophils in CLL. While their conventional antimicrobial functions decline, neutrophils are simultaneously reshaped by the leukemic microenvironment into cells that actively sustain the malignant clone. In CLL, neutrophils acquire an immunosuppressive phenotype resembling that of granulocytic myeloid-derived suppressor cells (G-MDSCs), a process recently termed the “neutrophil extracellular reprogramming signature” (NERST) [27]. In humans, this subset is characterized by a CD11b^+^CD15^+^CD66b^+^CD16^low/−^HLA-DR^−^ phenotype, often accompanied by CD62L shedding and increased CXCR4 expression. Functionally, these cells exhibit elevated levels of ARG1, PD-L1, and reactive oxygen species (ROS), contributing to T-cell suppression. These reprogrammed neutrophils, in turn, bolster leukemia cell persistence by delivering pro-survival signals via mediators such as BAFF and APRIL and by activating NF-κB, PI3K/AKT/mTOR pathways. Additionally, they suppress T-cell immunity by upregulating inhibitory checkpoint ligands such as PD-L1 and by releasing neutrophil extracellular traps (NETs), which can directly trigger T-cell apoptosis [27].

Collectively, these abnormalities compromise the frontline innate defense against bacterial and fungal pathogens. When combined with neutropenia—whether arising from the disease itself or from treatment—these defects markedly heighten the risk of serious infections, including severe bacterial pneumonias.

### 2.4. Impact of Modern Therapies on the Infection Profile

The introduction of targeted therapies has shifted the infection landscape in CLL. Although the decline in chemoimmunotherapy use has reduced the incidence of classical opportunistic infections, new patterns of infectious risk have emerged with Bruton tyrosine kinase inhibitors (BTKis), BCL2 inhibitors, and anti-CD20 monoclonal antibodies (Table 2) [28].

BTK inhibitors, including ibrutinib, acalabrutinib, and zanubrutinib, disrupt not only B-cell receptor signaling but also important signaling pathways in various immune effector cells. These drugs have been associated with an increased incidence of bacterial pneumonias and, in certain high-risk individuals, invasive fungal infections such as aspergillosis [29,30].

In a recent meta-analysis, Buchrits et al. evaluated a broad spectrum of infection-related outcomes in the CLL therapy landscape. BTKi monotherapy was not associated with a statistically significant increase in the risk of any infection (RR 1.12, 95% CI: 0.94–1.34) or grade 3–4 infections (RR 1.05, 95% CI: 0.76–1.44) when compared with chemotherapy-based regimens [31]. BTKi combinations with anti-CD-20 antibodies or venetoclax did not elevate infection risk [32]. However, the results of the phase III trial comparing finite treatment (Ibrutinib–venetoclax or venetoclax–obinutuzumab) versus treatment until progression with Ibrutinib have recently been published. In the venetoclax–obinutuzumab group, the percentage of patients with serious infections was higher than in the other groups [33].

Although the absolute risk remains lower than with agents such as fludarabine or alemtuzumab, the article by Martino et al. highlights the need for heightened awareness and individualized monitoring strategies in patients receiving BTKs [18].

Venetoclax, a potent BCL2 inhibitor, is frequently associated with severe and prolonged neutropenia, which constitutes one of the strongest risk factors for infection. Bacterial infections, sepsis, and respiratory viral infections are common complications, particularly during the dose-escalation phase or when venetoclax is combined with anti-CD20 antibodies [32]. Growth factor support and careful dose modifications are essential components of infection prevention in this setting.

Anti-CD20 antibodies, including rituximab and obinutuzumab, induce prolonged B-cell depletion and persistent hypogammaglobulinemia. These effects predispose to recurrent bacterial infections and viral reactivation, including hepatitis B and community respiratory viruses [13]. In selected patients with severe hypogammaglobulinemia or recurrent infections, immunoglobulin replacement therapy (IgRT) has been shown to reduce infection frequency and improve quality of life [34]. However, there is controversy surrounding the generalization of this recommendation.

### 2.5. Prevention Strategies and Clinical Implications

Given the complexity of immune dysfunction in CLL, infection prevention requires a personalized, multimodal approach. Current strategies include antiviral prophylaxis in patients receiving BTKis or anti-CD20 therapy, prophylaxis against *Pneumocystis jirovecii* pneumonia (PJP) in cases of intensive or prolonged immunosuppression, and close monitoring for fungal infections in selected high-risk patients [35]. Vaccination is strongly recommended, ideally before treatment initiation, despite suboptimal responses. IgRT should be considered for patients with recurrent bacterial infections and documented hypogammaglobulinemia [36,37].

Overall, although modern therapies have markedly improved survival in CLL, immune dysfunction remains a defining feature of the disease and a major driver of morbidity. The evolving spectrum of infections in the targeted-therapy era underscores the importance of individualized risk assessment, proactive prevention, and ongoing vigilance to minimize infectious complications and improve long-term outcomes.

## 3. Autoimmune Cytopenias

Autoimmune cytopenias (AIC) are among the most characteristic immune-mediated complications of CLL and represent a direct clinical consequence of the profound immune dysregulation associated with this disease. Unlike cytopenias related to bone marrow infiltration or treatment toxicity, AIC arises from immune tolerance loss and may occur at any stage of the disease, including before diagnosis or in patients with otherwise indolent disease [38]. Their recognition is essential, as they require a distinct diagnostic approach and therapeutic strategy.

### 3.1. Epidemiology and Clinical Timing

The reported incidence of AIC in CLL varies across series, ranging from approximately 4% to more than 10%, depending on diagnostic criteria and follow-up duration. Autoimmune hemolytic anemia (AIHA) is the most common manifestation, accounting for nearly two-thirds of cases, followed by immune thrombocytopenia (ITP) [39]. Pure red cell aplasia (PRCA) and autoimmune neutropenia are considerably rarer (less than 1%) [40]. Importantly, AIC may precede the diagnosis of CLL, coincide with initial presentation, or develop during the disease course, including during active therapy [19]. This temporal heterogeneity underscores that AIC are not simply a marker of advanced disease but rather a manifestation of underlying immune imbalance intrinsic to CLL biology. The occurrence of AIC is often associated with CLL at advanced stage; this supports the idea that the extensive immunologic alterations observed in actively progressive leukemia are at least in part responsible for the autoimmune phenomenon.

Among CLL patients, those with trisomy 12 are more likely to develop autoimmune cytopenias. It has been reported that up to 24% of these patients will develop this condition during the course of their disease [41,42].

### 3.2. Pathophysiology of Autoimmune Cytopenias in CLL

The pathogenesis of AIC in CLL is multifactorial and involves complex interactions between malignant B cells, non-malignant immune cells, and the leukemic microenvironment [43].

CLL cells themselves rarely produce pathogenic autoantibodies; instead, they act as aberrant antigen-presenting cells that stimulate polyclonal, non-malignant B cells to generate autoantibodies directed against blood cell antigens. In parallel, profound T-cell dysregulation plays a central role. Alterations in the balance between regulatory T cells and pro-inflammatory T-helper subsets, particularly Th17 cells, contribute to the breakdown of peripheral tolerance [44].

Cytokines secreted by CLL cells and stromal elements, including IL-10, TGF-β, and BAFF, further promote immune deviation and sustain autoreactive clones. The leukemic microenvironment thus serves as a permissive niche that amplifies and sustains autoimmunity. These mechanisms explain why AIC may persist or recur even when tumor burden is modest and why immunosuppressive therapies alone may be insufficient in some cases.

### 3.3. Autoimmune Hemolytic Anemia

AIHA is the most common autoimmune cytopenia associated with CLL. It is typically mediated by warm-reactive IgG autoantibodies directed against red blood cell antigens, leading to extravascular hemolysis. Clinically, patients may present with anemia, jaundice, elevated lactate dehydrogenase, indirect hyperbilirubinemia, and reduced haptoglobin levels. A direct antiglobulin test (DAT) is positive in most cases, although DAT-negative AIHA may occur and requires careful clinical correlation [45].

The development of AIHA does not necessarily correlate with CLL stage or tumor burden. Several studies suggest an association with adverse biological features, such as unmutated IGHV status or certain cytogenetic abnormalities, although findings are not entirely consistent. First-line treatment generally consists of corticosteroids, which induce responses in a substantial proportion of patients. In steroid-refractory or dependent cases, rituximab-based regimens are commonly employed, and treatment of the underlying CLL is considered when immune-directed therapy fails [46].

### 3.4. Immune Thrombocytopenia

ITP represents the second most frequent AIC in CLL and is characterized by immune-mediated platelet destruction in the absence of significant bone marrow failure. Diagnosis is challenging and requires exclusion of marrow infiltration, hypersplenism, and treatment-related thrombocytopenia. Bone marrow examination typically reveals preserved or increased megakaryocytes, supporting a peripheral mechanism of platelet loss [47].

Clinically significant bleeding is less common than in primary ITP but may occur, particularly in elderly patients or those receiving anticoagulation. Management parallels that of AIHA, with corticosteroids as first-line therapy and rituximab or other immunomodulatory approaches for refractory disease. Thrombopoietin receptor agonists have been used in selected cases, although data in CLL-associated ITP remain limited [48].

### 3.5. Pure Red Cell Aplasia and Other Rare Cytopenias

PRCA is an uncommon but well-recognized complication of CLL, characterized by severe normocytic anemia, reticulocytopenia, and a near-complete absence of erythroid precursors in the bone marrow. The condition is thought to be primarily T-cell-mediated rather than antibody-driven. Treatment often requires prolonged immunosuppression, and responses may be slow and incomplete. Autoimmune neutropenia is rare and should be diagnosed only after careful exclusion of other causes of neutropenia, including drug toxicity and marrow infiltration [49].

### 3.6. Impact of Modern Targeted Therapies

The introduction of targeted therapies has significantly altered the landscape of AIC in CLL. Effective control of the leukemic clone with agents such as BTK inhibitors or BCL2 inhibitors may lead to improvement or resolution of autoimmune cytopenias in some patients, supporting the concept that CLL-directed therapy can indirectly restore immune balance.

Ibrutinib, in particular, has been associated with durable responses in refractory AIC, likely due to its immunomodulatory effects on B-cell receptor signaling and T-cell function [50]. Treatment-emergent autoimmune hemolytic anemia (AIHA) during BTK inhibitor (BTKi) therapy in CLL is uncommon, with an incidence of approximately 1% in patients treated with ibrutinib (5–13 cases per 1000 patient-years) [50,51]. Available data suggest that these events more often reflect underlying disease activity rather than a direct drug-induced effect. In fact, BTKi therapy is more frequently associated with improvement of preexisting autoimmune cytopenias than with their induction. Reported rates vary across cohorts, with up to 6% in real-world series, typically occurring early after treatment initiation (median ~2 months) [52].

Treatment-emergent events are predominantly observed in patients with adverse biological features, including unmutated IGHV and TP53 disruption [50]. Mechanistically, BTKi-mediated ITK inhibition promotes Th1 polarization and partial immune rebalancing, which may explain their overall protective effect against autoimmunity. When AIHA occurs, standard diagnostic evaluation is required, and management includes corticosteroids and other immunosuppressive approaches. Importantly, BTKi therapy can be safely continued in most cases, with 64–83% of patients maintaining treatment [52]. Moreover, in patients with preexisting AIHA, up to 80% experience improvement, supporting BTKis as a preferred therapeutic strategy in this setting.

Table 3 summarizes the incidence and clinical course of autoimmune cytopenias (AICs) in patients with CLL before and during venetoclax treatment. Although most patients experience clinical improvement after starting targeted therapy, there is a group of patients who present with pre-existing AICs caused by disease-related immune dysregulation. Venetoclax is associated with a higher rate of treatment-emergent AICs compared to other targeted agents, although these events remain manageable in most cases and rarely require treatment interruption. Most of these autoimmune cytopenias tend to occur in patients with adverse biological features, suggesting a strong association with the disease’s biology. Despite this, venetoclax also induces partial immune reconstitution, reflecting a complex interplay between immune recovery and persistent dysregulation. Overall, these findings underscore the dual role of venetoclax in modulating autoimmunity in CLL.

Distinguishing treatment-induced cytopenias from true autoimmune phenomena remains a clinical challenge and requires careful longitudinal assessment.

### 3.7. Therapeutic Strategies

Management of AIC in CLL should be individualized and guided by the severity of cytopenia, presence of symptoms, and activity of the underlying leukemia. Corticosteroids remain the cornerstone of initial therapy, while rituximab is widely used in refractory or relapsing cases. Cyclosporine A (CyA) is an effective immunosuppressant for refractory cases, with reported response rates of approximately 80% in patients who have received previous intensive treatments. It acts by inhibiting T-cell activation and interleukin-2 production. It is particularly useful in allowing a gradual reduction in steroids and other agents, such as TPO receptor agonists, due to its steroid-sparing effects [53].

In patients with persistent or recurrent AIC, treatment of CLL with targeted agents may be necessary even in the absence of classic indications for therapy. Splenectomy is now rarely performed and reserved for highly selected cases.

### 3.8. Prognostic Implications

The prognostic significance of AIC in CLL remains controversial. While some studies associate AIC with adverse disease features, others suggest that when appropriately treated, AIC does not independently worsen overall survival. Importantly, cytopenias of autoimmune origin should not be equated with advanced-stage disease, as this may lead to inappropriate staging and treatment decisions.

Autoimmune cytopenias represent a clinically important manifestation of immune dysfunction in CLL. Their pathogenesis reflects the intricate interplay between malignant B cells, dysregulated T-cell responses, and a permissive microenvironment. Advances in targeted therapy have improved the management of both CLL and its autoimmune complications. However, optimal care requires accurate diagnosis, careful differentiation from other causes of cytopenia, and an integrated therapeutic approach.

## 4. Secondary Neoplasms in Patients with Chronic Lymphocytic Leukemia

CLL is a disease characterized by profound immune dysregulation, which predisposes patients not only to infections and autoimmune phenomena but also to the development of second primary malignancies (SPMs) and other secondary neoplastic conditions. The recognition of this increased risk has evolved from early case reports to large cohort and registry studies, demonstrating that patients with CLL carry a significantly elevated likelihood of developing additional cancers compared with age-matched controls in the general population. This phenomenon encompasses a wide spectrum of neoplasms, including second hematological malignancies, solid tumors, skin cancers, and disease transformations such as Richter’s syndrome (RS) (the latter frequently discussed under the framework of multiple B-cell malignancies in CLL).

### 4.1. Epidemiology of Secondary Malignancies in CLL

Numerous epidemiological studies and cancer registry analyses have documented the increased incidence of SPMs in CLL patients. Compared with the general population, CLL is associated with a markedly higher risk of second cancers. This finding was first recognized decades ago and has been consistently confirmed in more contemporary cohorts.

Patients with CLL have a consistently higher risk of secondary primary neoplasms (SPNs), with standardized incidence ratios (SIRs) ranging from 1.14 to 2.17 across different populations. Large registry-based studies show an overall SIR of 1.20 (95% CI: 1.17–1.23) in SEER, 1.63 (95% CI: 1.59–1.68) in the Netherlands, and up to 2.17 (95% CI: 2.07–2.27) in Australia, reflecting geographic and environmental variability. The excess risk is more pronounced for hematologic neoplasms (SIR up to 1.61) compared with solid tumors (SIR ~1.15–1.67). Specific tumor types show markedly elevated relative risks, particularly non-melanoma skin cancers and virus-associated neoplasms, with relative risks exceeding 7–24 in some cohorts. Patient-related factors, such as age > 65 years (HR 2.1), male sex (HR 1.7), and multiple lines of treatment (HR up to 12.1), further contribute to the risk of SPN. These data underscore the multifactorial nature of secondary malignancies in CLL, driven by immune dysfunction, treatment exposure, and host-related factors [54,55].

A large retrospective study encompassing multiple eras of CLL therapy found that among a cohort of over 500 patients with CLL or small lymphocytic lymphoma (SLL), nearly one-third developed at least one SPM during follow-up. Skin cancers, solid organ malignancies, second hematological malignancies, and aggressive histologic transformations such as RS were documented [56,57,58] (Table 4).

### 4.2. Types of Secondary Neoplasms

#### 4.2.1. Skin Cancers

Cutaneous malignancies are among the most prevalent SPMs in CLL. Both melanoma and non-melanoma skin cancers (NMSCs) occur at increased rates, with certain series reporting that a substantial percentage of CLL patients eventually develop skin cancer. Interestingly, the typical ratio of basal cell carcinoma to squamous cell carcinoma may be altered in CLL, with squamous cell carcinoma occurring disproportionately more often, including cases with metastatic potential [59,60,61].

#### 4.2.2. Solid Organ Malignancies

Solid tumors—including prostate, breast, lung, and gastrointestinal cancers—are also significantly more common in CLL patients than in the general population. Data indicate that a variety of epithelial malignancies contribute to the burden of SPMs, and their incidence increases with age and cumulative exposure to cancer therapeutic strategies [54,62].

#### 4.2.3. Second Hematological Malignancies

In addition to CLL itself, patients may develop other hematologic cancers, including therapy-related myeloid neoplasms (such as acute myeloid leukemia (AML) and myelodysplastic syndromes (MDSs)). While these events are less frequent than solid organ tumors or skin cancers, they are of particular clinical concern because they often carry poor prognoses. Some studies report that up to 5–6% of CLL patients develop a second hematological malignancy during long-term follow-up, and a subset of these cases are thought to be therapy-related [60].

#### 4.2.4. Richter’s Syndrome and Multiple B-Cell Malignancies

One of the most impactful secondary neoplasms in CLL is RS, a transformation of CLL into an aggressive lymphoma, most commonly diffuse large B-cell lymphoma (DLBCL), and less often into Hodgkin lymphoma. RS represents not merely a new cancer but a biological transformation of the original neoplastic clone in many cases. It occurs in approximately 2–10% of CLL patients, often abruptly during the disease course, and is characterized by rapid clinical deterioration [63].

Clonally unrelated B-cell malignancies have also been described, in which CLL patients develop additional lymphoid neoplasms with distinct clonal origins. These rare events underscore the complex genomic instability inherent to CLL biology and the ongoing risk of multiple neoplasms.

### 4.3. Pathophysiology of Secondary Neoplasms in CLL

The pathogenesis of secondary neoplasms in patients with CLL is complex and multifactorial, reflecting the interaction between intrinsic disease biology, profound immune dysregulation, and the cumulative effects of therapy. Rather than being incidental events, secondary malignancies arise within a biologically permissive environment characterized by impaired tumor immune surveillance, chronic inflammation, genomic instability, and selective pressures imposed by treatment.

#### 4.3.1. Impaired Immune Surveillance and Failure of Tumor Control

One of the central mechanisms predisposing CLL patients to secondary cancers is the sustained impairment of immune surveillance. CLL is associated with deep defects across both adaptive and innate immune compartments. Hypogammaglobulinemia and dysfunctional antibody production compromise humoral immunity, while T-cell abnormalities—including skewed CD4/CD8 ratios, reduced cytotoxic capacity, impaired synapse formation, and features of functional exhaustion—limit effective antitumor responses. Overexpression of inhibitory immune checkpoints such as PD-1, CTLA-4, and LAG-3 further suppresses cellular immunity [15,64].

Natural killer (NK) cells, which play a critical role in immune-mediated tumor elimination, also exhibit reduced cytotoxic activity and impaired signaling in CLL. Collectively, these defects weaken the immune system’s ability to detect and eliminate emerging malignant clones, facilitating the development of both hematologic and solid secondary neoplasms.

This failure of immune surveillance is particularly relevant for malignancies in which immune control plays a dominant role, such as virus-associated cancers and cutaneous tumors. The disproportionately high incidence and aggressive behavior of squamous cell carcinoma of the skin in CLL patients strongly support this mechanism.

#### 4.3.2. Chronic Antigenic Stimulation and Pro-Oncogenic Inflammatory Milieu

CLL is characterized by chronic antigenic stimulation and persistent immune activation within lymphoid tissues. Continuous interactions among leukemic B cells, T cells, and stromal elements generate a cytokine-rich microenvironment, dominated by IL-6, IL-10, TNF-α, BAFF, and APRIL. While this milieu supports leukemic cell survival and proliferation, it simultaneously promotes immune dysfunction and chronic inflammation [65].

Sustained inflammatory signaling can induce oxidative stress, DNA damage, and aberrant cellular proliferation in both hematopoietic and non-hematopoietic cells. Over time, this environment may facilitate malignant transformation at distant sites, contributing to the increased incidence of solid tumors and secondary hematologic malignancies observed in CLL populations.

#### 4.3.3. Genomic Instability and Clonal Evolution

Intrinsic genomic instability is a hallmark of CLL and represents another key contributor to secondary neoplasm development. Defects in DNA damage response pathways—particularly those involving TP53 and ATM—not only drive disease progression and therapeutic resistance but also reflect a broader susceptibility to malignant transformation.

In recent years, clonal hematopoiesis of indeterminate potential (CHIP) has emerged as an important biological link between CLL, aging, and therapy-related malignancies. CHIP-associated mutations, frequently involving epigenetic regulators such as DNMT3A, TET2, and ASXL1, are increasingly detected in CLL patients, especially those exposed to cytotoxic therapy. These pre-existing clones may expand under treatment-induced selective pressure, ultimately giving rise to therapy-related myeloid neoplasms such as myelodysplastic syndromes and acute myeloid leukemia [15,59].

#### 4.3.4. Treatment-Related Oncogenesis

Historically, exposure to alkylating agents and purine analogs has been strongly associated with the development of secondary malignancies in CLL. These therapies induce cumulative DNA damage in hematopoietic progenitor cells, increasing the risk of therapy-related myeloid neoplasms characterized by complex cytogenetics and dismal outcomes.

Although the transition to targeted therapies has reduced reliance on genotoxic chemotherapy, the long-term oncogenic consequences of sustained pathway inhibition remain incompletely defined. Targeted agents may influence immune homeostasis, DNA repair mechanisms, and clonal selection dynamics. Importantly, effective disease control with modern therapies may partially restore immune competence, potentially mitigating some aspects of secondary cancer risk, although long-term observational data are still needed.

In trials such as ELEVATE-TN, the incidence of second primary malignancies was found to be generally consistent or similar between acalabrutinib-containing arms and obinutuzumab–chlorambucil. While BTK inhibitors like acalabrutinib can be associated with a risk of secondary malignancies, studies show a 3-year cumulative incidence of roughly 7% for SPMs (excluding NMSC) in patients receiving BTK inhibitors, with a higher risk observed in smokers or patients with low baseline CD8 counts [66].

The updated analysis with 6-year follow-up of the CLL14 clinical trial comparing two finite treatment regimens (Ven-Obi) vs. (Clb-Obi) indicates that the adjusted incidence of second primary malignancies was slightly higher in the Ven-Obi arm compared to Clb-Obi. However, this finding does not translate into a clear signal of a clinically relevant increase in treatment-attributable risk, especially given the observed benefits in progression-free survival and disease control with Ven-Obi [67].

#### 4.3.5. Disease Transformation and Multiple B-Cell Neoplasms

RS represents a unique and particularly aggressive form of secondary neoplasia in CLL. In the majority of cases, transformation reflects clonal evolution of the original leukemic clone through acquisition of additional genetic lesions, including TP53 disruption, MYC activation, and NOTCH1 mutations. In a minority of cases, Richter-like lymphomas are clonally unrelated, highlighting the susceptibility of CLL patients to de novo lymphoid malignancies in the context of immune dysfunction. An in-depth review of RS is outside the focus of this review [63].

Beyond RS, the occurrence of multiple clonally distinct B-cell neoplasms in CLL underscores the concept of CLL as a systemic disorder of immune dysregulation and genomic instability, rather than a disease confined to a single malignant clone.

### 4.4. Clinical Implications and Prognostic Impact

The development of a secondary neoplasm in a CLL patient often portends a worse overall prognosis, particularly in hematologic malignancies and RS. Many studies demonstrate that survival after a secondary cancer diagnosis is significantly reduced compared with that of CLL patients without SPM.

Skin cancers and solid tumors, although sometimes amenable to standard treatments, contribute to cumulative morbidity and mortality. Hematological secondary malignancies, including therapy-related AML/MDS and RS, remain particularly aggressive and may require intensive and specialized therapies.

### 4.5. Surveillance and Management Considerations

Given the heightened SPM risk, clinicians should maintain a high index of suspicion for new malignancies during routine follow-up of CLL patients. Appropriate cancer screening—including dermatologic evaluation, age-appropriate organ cancer screening, and vigilant assessment for symptoms suggestive of hematologic transformation—is recommended as part of comprehensive long-term care.

In patients treated with cytotoxic agents, especially those with prolonged exposure, more intensive monitoring for therapy-related myeloid neoplasms may be justified. Meanwhile, the adoption of targeted therapies that preserve immune function where possible may mitigate some SPM risk, though this remains an area of active investigation.

The NCCN CLL/SLL Guidelines specifically address cancer screening as part of supportive care, recommending annual dermatologic skin screening and strict adherence to age-appropriate cancer screening guidelines for breast, cervical, colon, and prostate cancers. Below (Table 5) is a practical, evidence-based screening protocol organized by neoplasm category [68].

## 5. Conclusions

Immune dysfunction represents a central and defining feature of chronic lymphocytic leukemia, extending far beyond a secondary consequence of tumor burden or treatment exposure. Profound alterations affecting both innate and adaptive immunity underpin the increased susceptibility to infections, the development of autoimmune cytopenias, and the elevated risk of secondary malignancies that collectively contribute to morbidity and mortality in CLL patients throughout the disease course.

Despite major therapeutic advances and improved disease control with targeted agents, immune dysregulation often persists and continues to shape clinical outcomes. The evolving infection landscape, the complex biology of autoimmune cytopenias, and the sustained risk of secondary neoplasms highlight the need for integrated management strategies that address not only leukemic eradication but also immune restoration and long-term surveillance.

A deeper understanding of the molecular and cellular mechanisms driving immune dysfunction in CLL may inform the development of more personalized preventive strategies and novel therapeutic approaches to restore immune competence. Ultimately, incorporating immune health as a core component of CLL management will be essential to further improve long-term outcomes and quality of life for patients.

## Figures and Tables

**Table 1:** Immune defects in CLL and associated clinical consequences.

Immune Compartment	Functional Defect	Clinical Consequences
B cells	Hypogammaglobulinemia and impaired antibody production	Recurrent bacterial infections; poor vaccine responses
T cells	CD4/CD8 imbalance; functional exhaustion; impaired cytotoxicity	Increased susceptibility to viral infections (HSV, CMV, VZV)
Neutrophils	Defective chemotaxis, phagocytosis, and oxidative burst	Impaired bacterial clearance; increased risk of fungal infections
Natural killer cells	Reduced cytotoxic activity; impaired immune synapse formation	Viral reactivation (e.g., EBV, CMV)
Complement system	Deficiency of the classical complement pathway	Increased susceptibility to infections by encapsulated bacteria

**Table 2:** Infection risk profile according to treatment modality in chronic lymphocytic leukemia.

Treatment Modality	Predominant Infections	Key Considerations
Watch and wait	Respiratory tract infections	Predominantly bacterial; associated with hypogammaglobulinemia
FCR (fludarabine, cyclophosphamide, rituximab)	Bacterial, viral (CMV), fungal, Pneumocystis jirovecii pneumonia	Profound and prolonged lymphopenia and neutropenia
BTK inhibitors	Bacterial pneumonia; invasive fungal infections (e.g., aspergillosis)	Increased early infection risk, particularly in previously treated patients
Venetoclax + anti-CD20 antibodies	Bacterial, viral and fungal infections	Risk increases during treatment-related neutropenia
Anti-CD20 monoclonal antibodies	Viral reactivation (HBV, VZV)	Long-lasting B-cell depletion and impaired humoral immunity

**Table 3:** Autoimmune cytopenias (AICs) before and during venetoclax therapy in CLL.

Parameter	Before Venetoclax (Preexisting AICs)	During Venetoclax (Treatment-Emergent AICs)
**Prevalence/Incidence**	~13% of CLL patients had a history of AICs	~7% develop AICs during treatment
**Clinical behavior**	Often persistent at treatment initiation	Typically occur early during therapy
**Response to therapy**	~80% show improvement or resolution under targeted therapy	Majority manageable; do not preclude continuation
**Incidence rate**	Not applicable	~69 episodes per 1000 patient-years
**Comparison with other agents**	Not applicable	Higher than ibrutinib (~5/1000 pt-years) and idelalisib (~6/1000 pt-years)
**Biological profile**	Associated with underlying immune dysregulation	Predominantly in high-risk patients (unmutated IGHV, del(17p)/TP53)
**Management**	Often improves with CLL-directed therapy	83% continue venetoclax ± immunosuppressive treatment
**Clinical interpretation**	Reflects CLL-driven autoimmunity	May reflect both disease biology and treatment-related effects
**Mechanistic context**	Immune dysregulation driven by CLL clone	Partial immune rebalancing (↓ Tregs, ↓ PD-1^+^ CD8^+^, ↑ NK function)
**Therapeutic implication**	Supports use of targeted therapy	BTKis may be preferred when AIC is the primary indication

**Table 4:** Risk of second primary neoplasms in CLL: SIR, RR and HR by tumor type and risk factors.

Category	Neoplasm/Factor	Risk Estimate (95% CI)	Population/Study
**Overall risk**	Any SPN	SIR 1.20 (1.17–1.23)	SEER (USA)
		SIR 1.63 (1.59–1.68)	Netherlands
		SIR 2.17 (2.07–2.27)	Australia
		SIR 1.14 (1.00–1.29)	Taiwan
**Hematologic neoplasms**	Any hematologic malignancy	SIR 1.61 (1.50–1.73)	SEER
		SIR 1.42 (1.24–1.62)	Netherlands
	Hodgkin lymphoma	RR 7.16	Sweden
		O/E 7.69	SEER
	MDS/AML (post FC ± R)	OR 3.7 (2.79–4.91)	ERIC/HARMONY
	MDS (fludarabine)	HR 4.93 (1.2–19.8)	Denmark
**Skin cancers**	SCC in situ	RR 24.58	Sweden
	Invasive SCC	RR 7.63	Sweden
	Merkel cell carcinoma	RR 14.36	Sweden
	Melanoma	SIR 7.74 (6.85–8.72)	Australia
		O/E 3.18	SEER
	Melanoma (ibrutinib)	SIR 15.8 (7.0–35.3)	Australia
	Non-melanoma skin cancer	SIR 2.55 (1.60–3.86)	Taiwan
**Virus-related tumors**	Kaposi sarcoma	RR 6.76	Sweden
		O/E 5.09	SEER
**Solid tumors**	Lung	O/E 1.66–1.90	SEER
	Larynx	O/E 1.72	SEER
	Brain	O/E 1.98	SEER
	Bladder	SIR 1.99 (1.06–3.41)	Taiwan
	Stomach (women)	O/E 1.76	SEER
**Risk factors (HR)**	Age > 65 years	HR 2.1	GCLLSG
	Male sex	HR 1.7	GCLLSG
	Comorbidities	HR 1.6	GCLLSG
	≥1 prior treatment line	HR 12.1	GCLLSG
**Treatment-related risk**	Chemotherapy exposure	HR 1.51 (1.3–1.8)	Denmark
	Targeted therapy (NMSC)	OR 1.8 (1.36–2.41)	ERIC/HARMONY
	Prostate cancer (treated)	OR 2.11 (1.12–3.97)	ERIC/HARMONY

**Table 5:** NCCN recommendations for SPM screening in CLL patients.

Screening Domain	Recommendation	Frequency
Dermatologic exam	Full-body skin exam by dermatologist; low biopsy threshold	Annually (q6 months if high-risk)
Sun protection counseling	SPF ≥ 30, UV avoidance education	Every visit
Breast cancer	Mammography per standard guidelines	Per USPSTF/ACS guidelines
Cervical cancer	Pap/HPV testing per standard guidelines	Per USPSTF/ACS guidelines
Colorectal cancer	Colonoscopy per standard guidelines	Per USPSTF/ACS guidelines

## Data Availability

Data sharing is not applicable to this article as no new data were generated or analyzed.
